# The alert for emergence of pig-associated MRSA ST 398 in multi-regions China

**DOI:** 10.1016/j.onehlt.2025.101132

**Published:** 2025-07-17

**Authors:** Mingquan Cui, Hejia Wang, Ningning Han, Wenli Tang, Xiangbin Song

**Affiliations:** a China Institute of Veterinary Drug Control, Beijing, China; b Key Laboratory of Animal Antimicrobial Resistance Surveillance, Ministry of Agriculture and Rural Affairs, China; c Food and Agriculture Organization of the United Nations Antimicrobial Resistance Reference Center, China; d Shandong Center for Quality Control of Feed and Veterinary Drug, China; e Shandong Provincial Key Laboratory of Quality Safety Monitoring for Animal Products and Veterinary Drug Innovation, Ji'nan, Shandong Province 250100, China

**Keywords:** Pig-associated *Staphylococcus aureus*, MRSA ST398, Antimicrobial resistance, Molecular epidemiology

## Abstract

Livestock-associated Methicillin-resistant *Staphylococcus aureus* (LA-MRSA) lineage ST398 presents a notable public health risk through its transmission from pigs to humans. This study characterizes phenotypic and genomic features of pig-associated MRSA ST398 isolates in China. Through surveillance (2015–2021) of 296 *S. aureus* isolates from major pork-producing regions, we identified 14 MRSA isolates in Zhejiang, Fujian, Guangdong, and Shanxi provinces, with 12 (85.7 %) belonging to the ST398 lineage. These isolates displayed two genetic profiles: t011-SCC*mec* V (10/12) and t571-SCC*mec* XII (2/12), and exhibited multidrug resistance to penicillin, oxacillin, cefoxitin, erythromycin, and clindamycin. Phylogenetic analysis revealed three critical findings: (1) clonal dissemination within Shanxi (0–48 SNPs), (2) potential interprovincial spread between geographically distant regions (Shanxi-Qinghai, 17–44 SNPs), and (3) concerning genetic linkage (70 SNPs) between a Zhejiang isolate and European ST398 isolate. These results underscore the urgent need for integrated pork supply chain surveillance strategies to monitor this emerging zoonotic pathogen.

## 1 Introduction

*Staphylococcus aureus*, particularly methicillin-resistant *Staphylococcus aureus* (MRSA), represents an opportunistic zoonotic pathogen capable of causing diverse clinical manifestations ranging from superficial skin infections to life-threatening conditions such as necrotizing pneumonia and toxic shock syndrome across Europe [1]. MRSA poses a particularly serious challenge in clinical settings due to its multidrug resistance, driven by virulence factors such as toxic shock syndrome toxin-1 (TSST-1), Panton-Valentine leucocidin (PVL), Von Willebrand factor-binding protein (vWbp), and staphylococcal enterotoxins [2]. Among MRSA strains, the ST398 lineage presents a notable public health risk, with pigs identified as its primary reservoir [3]. Zoonotic transmission of MRSA ST398 from pigs to humans has prompted global efforts to assess its potential threat [4]. While MRSA ST9 predominates in Chinese swine populations [5], MRSA ST398 is more commonly associated with pigs in Europe and North America [6]. Previous studies on livestock-associated methicillin-resistant *Staphylococcus aureus* (LA-MRSA) in China have demonstrated that the ST398 isolate was relatively uncommon in swine populations, with only sporadic detections reported in Guangdong and Shandong provinces [7,8]. However, recent surveillance data have revealed a concerning emergence of ST398 as a predominant source of MRSA infections among pigs at both farm and slaughterhouse levels in Qinghai province [9]. This epidemiological shift has raised significant public health concerns regarding the potential dissemination of MRSA ST398 through China's vast pork distribution networks. Given China's position as the world's largest pork producer, understanding the epidemiology of MRSA ST398 at the human-animal interface is critical for developing effective containment strategies. This study is one of the first to provide a comprehensive genomic and epidemiological characterization of MRSA ST398's geographical distribution, antimicrobial resistance profiles, and transmission dynamics within China's pork production system.

## 2 Materials and methods

### 2.1 Identification and susceptibility profiles of MRSA

We investigated the resistance phenotype data of 296 pig-associated *S. aureus* isolates from 11 provinces in China from 2015 to 2021, which were stored in the China Antimicrobial Resistance Surveillance Laboratory for Animal-Derived Bacteria Data Center. The 296 pig-associated *S. aureus* were isolated from 464 nasal swabs and 1293 faecal samples collected from randomly selected individual pigs in 33 pig farms and 12 slaughterhouses. MRSA isolates of with resistance phenotype were presented in four positive provinces where 38 *S. aureus* were isolated from 173 porcine samples. Then, the MRSA isolates were confirmed by phenotypic antibiotic resistance and the presence of *mecA* and *mecC* genes using Illumina sequencing.

Antimicrobial susceptibility of the 14 MRSA isolates was assessed using the broth dilution method. The tested antibiotics included penicillin, oxacillin, cefoxitin, gentamicin, trimethoprim/sulfamethoxazole, sulfisoxazole, clindamycin, erythromycin linezolid, florfenicol and ofloxacin, with results interpreted according to Clinical and Laboratory Standards Institute (CLSI) recommendations [10]. *S. aureus* ATCC 29213 was used in assays as the quality control strain. The bacterial isolate was considered multidrug-resistant (MDR) when it showed resistance to three or more classes of antimicrobial agents.

### 2.2 Whole genome sequencing and bioinformatics analysis

Genomic DNA of MRSA isolates was extracted from overnight cultures grown at 37 °C using TIANamp Bacteria DNA Reagent Kit (Tiangen, Beijing, China). Whole genomes of the MRSA isolates were sequenced by the Illumina HiSeq 2500 system and then assembled by SPAdes Genome Assembler [11]. Antimicrobial resistance genes and virulence genes were identified using modules Resfinder and VFDB, respectively, in ABRicate [12,13]. MLST was conducted using the SRST2 toolkit [14]. SCC*mec* and *spa* typing were conducted using SCC*mec* Finder and *spa* Typer in Center for Genomic Epidemiology (CGE) Services. (https://cge.cbs.dtu.dk/services/). To determine the evolutionary relationships among MRSA ST398 isolates from human and pig, an additional 32 human and livestock associated MRSA ST398 genome sequences (Table S1) were selected from MRSA metadata previously published in the literatures [9,15,16]. Snippy (https://github.com/tseemann/snippy) was used to do the genome alignment of the 44 MRSAST398 isolates, with the isolate S0385 as the reference. Putative recombinant regions were removed by Gubbins (https://github.com/nickjcroucher/gubbins). Core genome single nucleotide polymorphisms (SNPs) analysis were analyzed by Harvest [17] and IQ-TREE web server (http://iqtree.cibiv.univie.ac.at/).

## 3 Results and discussion

### 3.1 Prevalence of MDR MRSA ST398 in Chinese swine production

**Fig. 1:**
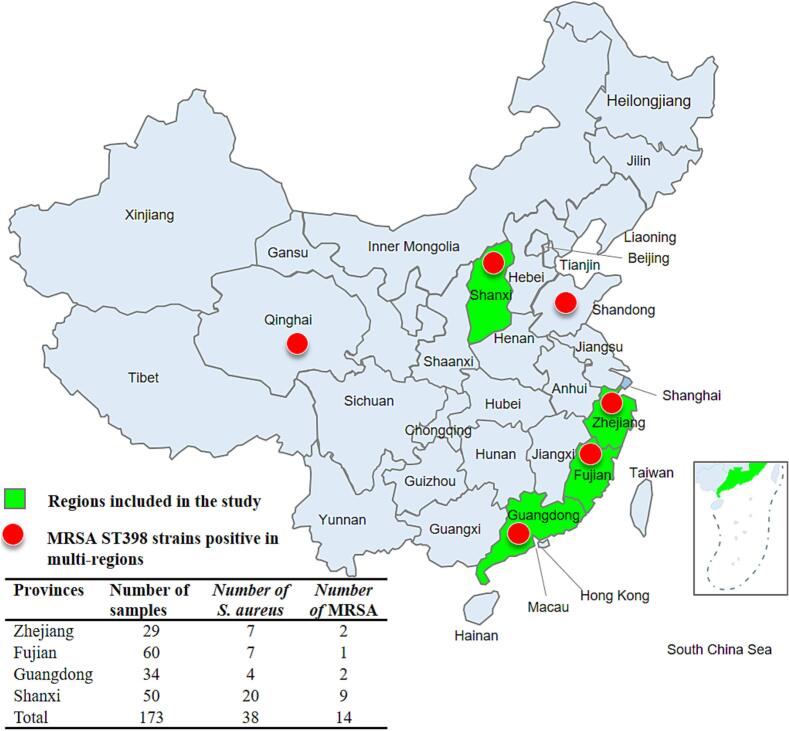
Geographical distribution of sample collection areas in China. Each label shows the province, sample size and the number of obtained *S. aureus* and MRSA isolates.

Among 38 *S. aureus* isolates isolated from 173 porcine samples across four provinces (Zhejiang, Fujian, Guangdong, Shanxi), 14 were confirmed as MRSA through phenotypic resistance profiling and *mecA*/*mecC* detection (Fig. 1). Antibiotic resistance profiles and genomic characterization of these pig-associated MRSA isolates were summarized in Table 1. All MRSA ST398 isolates carrying *mecA* gene exhibited multidrug resistance (MDR) to β-lactams (penicillin, oxacillin, cefoxitin), macrolides (erythromycin), and lincosamides (clindamycin), consistent with resistance patterns observed in European animal-derived MRSA ST398 isolates [18].

**Table 1:** Phenotypic and genomic characterization of the 14 pig-associated MRSA isolates.

Isolates	Provinces	MLST	SCC*mec*	*spa*	Resistance patterns	Antibiotic resistance profile
46–2019	Zhejiang	9	XII	t2922	PEN-ENR-CLI-OFX-FOX-OXA-FFC	*mecA*, *erm(C), blaZ, fexA, tet(K), tet(M), aac(6′)-aph(2″), ant(6)-Ia, dfrG, lnu(B)*
47–2019	Zhejiang	398	V	t011	PEN-ENR-CLI- FOX-OXA	*mecA*, *erm(C), blaZ, tet(K), tet(M), vga(A)*
48–2019	Fujian	398	XII	t571	PEN-ENR-CLI-OFX-FOX-OXA-GEN-FFC	*mecA*, *erm(T), blaZ, fexA, tet(L), aac(6′)-aph(2″), ant(6)-Ia, dfrG, lnu(B)*
57–2019	Guangdong	7191	V	t034	PEN-ENR-CLI- FOX-OXA	*mecA*, *erm(C),blaZ, tet(K), tet(M),lnu(B), dfrG), str*
58–2019	Guangdong	398	XII	t571	PEN-ENR-CLI-OFX-FOX-OXA-FFC	*mecA*, *erm(C), erm(T), blaZ, fexA, tet(L), aac(6′)-aph(2″), ant(6)-Ia, dfrG, lnu(B)*
174–2020	Shanxi	398	V	t011	PEN-ENR-CLI- FOX-OXA-GEN-FFC	*mecA*, *erm(C), blaZ, fexA, tet(K), tet(M), str*
175–2020	Shanxi	398	V	t011	PEN-ENR-CLI- FOX-OXA-FFC	*mecA*, *erm(C), blaZ, fexA, tet(K), tet(M), str*
176–2020	Shanxi	398	V	t011	PEN-ENR-CLI- FOX-OXA-FFC	*mecA*, *erm(C), blaZ, fexA, tet(K), tet(M)*
177–2020	Shanxi	398	V	t011	PEN-ENR-CLI- FOX-OXA-FFC	*mecA*, *erm(C), blaZ, fexA, tet(K), tet(M)*
178–2020	Shanxi	398	V	t011	PEN-ENR-CLI-OFX-FOX-OXA-FFC	*mecA*, *erm(C), blaZ, fexA, tet(K), tet(M)*
179–2020	Shanxi	398	V	t011	PEN-ENR-CLI- FOX-OXA-FFC	*mecA*, *erm(C), blaZ, fexA, tet(K), tet(M), str*
180–2020	Shanxi	398	V	t011	PEN-ENR-CLI- FOX-OXA-FFC	*mecA*, *erm(C), blaZ, fexA, tet(K), tet(M)*
181–2020	Shanxi	398	V	t011	PEN-ENR-CLI- FOX-OXA-FFC	*mecA*, *erm(C), blaZ, fexA, tet(K), tet(M)*
182–2020	Shanxi	398	V	t011	PEN-ENR-CLI- FOX-OXA-FFC	*mecA*, *erm(C), blaZ, fexA, tet(K), tet(M), str*

PEN, penicillin; OXA, oxacillin; FOX, cefoxitin; GEN, gentamicin; SXT, trimethoprim/sulfamethoxazole; SF, sulfisoxazole; CLI, clindamycin; ERY, erythromycin; OFX, ofloxacin; FFC, florfenicol.

### 3.2 Molecular epidemiology reveals high-risk lineages

Among the 14 MRSA isolates, three MLST types and four distinct *spa*-SCC*mec* combinations were detected. ST398 was dominant (12/14) and the remaining two isolates belonged to other STs (ST9 and ST7191). The predominant t011-SCC*mec* V lineage accounted for 71.4 % (10/14) of isolates, with geographic clustering observed in Shanxi (*n* = 9) and Zhejiang (*n* = 1). The t571-SCC*mec* XII was identified in two ST398 isolates from Guangdong and Fujian provinces, while the t2922-SCC*mec* XII and t034-SCC*mec* V were detected in single ST9 (Zhejiang) and ST7191 (Guangdong) isolates, respectively. Integrating the molecular characterization data from limited prior reports [[7], [8], [9]] and this investigation, we identified four pig-associated MRSA ST398 subtypes in China: t011-SCC*mec* V, t571-SCC*mec* XII, t034-SCC*mec* V, and t437-SCC*mec* XII. Critically, the t011-SCC*mec* V and t034-SCC*mec* V subtypes corresponded to clinical MRSA subtypes documented in human clinical infections [16,19], indicating significant zoonotic transmission risks within the One Health framework. These findings highlight the potential for occupational exposure among farm workers, slaughterhouse employees, and meat processing staff in China, emphasizing the urgent need for integrated surveillance bridging pig and human health sectors.

Notably, these pig-associated MRSA ST398 isolates shared similar antimicrobial resistance and virulence gene profiles with human-associated MRSA ST398 isolates from Europe [2]. All isolates carried characteristic resistance genes (*mecA*, *erm*, *tet*, *blaZ*), while exhibiting host adaptation through absence of human-specific virulence factors (PVL, TSST-1) but retention of Von Willebrand factor-binding protein (vWbp).

### 3.3 Genomic evidence of multiscale transmission

**Fig. 2:**
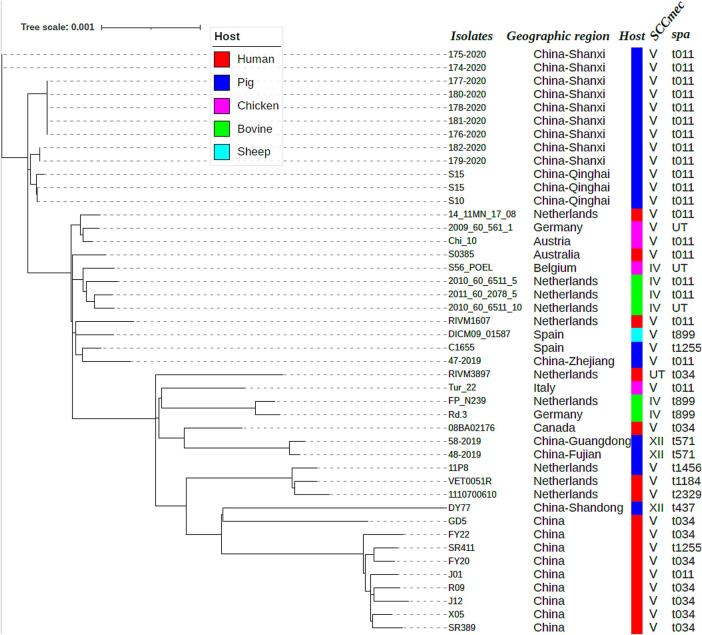
Phylogeny of 44 MRSA ST398, composed of 12 MRSA ST398 isolates in the present study and 32 additional MRSA ST398 available in the NCBI GenBank. “UT” indicates “untypeable”.

The draft genomes of all 14 MRSA isolates have been deposited in GenBank with accession number PRJNA 1240852. Phylogenetic analysis (Fig. 2) demonstrated close evolutionary relationships among twelve pig-associated MRSA ST398 isolates, including nine isolates from Shanxi province and three isolates from Qinghai province. Among them, the nine MRSA ST398 isolates from Shanxi exhibited minimal genetic divergence (0–48 SNPs), providing strong evidence for intra-farm clonal transmission base on a transmission threshold of ≤40 SNPs for *S. aureus* [20]. This observation aligns with previous reports of MRSA ST398 transmission within swine operations in Qinghai [7]. Notably, we observed significant genetic similarity (17–44 SNPs) between the Shanxi isolates and three MRSA ST398 isolates from geographically distant Qinghai province. Furthermore, Phylogenetic analysis revealed a distinct clade, phylogenetically close to human-associated MRSA ST398, which consisted of two isolates from adjacent provinces (Fujian and Guangdong) and exhibited 25 SNP differences. The close genetic relatedness of MRSA ST398 isolates across provinces suggests the possible interprovincial transmission of pig-associated MRSA ST398 in China. These findings demonstrate the dissemination dynamics of pig-associated MRSA ST398 in China, highlighting both localized clonal expansion and broader regional transmission risks. Of particular concern, the Zhejiang isolate showed striking phylogenetic proximity (70 SNPs differences) to porcine-associated MRSA ST398 isolates from Spain, underscoring the necessity for international surveillance frameworks to monitor antimicrobial resistance dissemination through global livestock trade routes.

## 4 Conclusion

A key limitation of this study is the relatively small number of MRSA isolates (*n* = 14), which may not fully represent the diversity or true prevalence of MRSA ST398 in China's swine industry. Broader sampling and integration of metadata (e.g., animal movement, human contact) would strengthen future analyses. However, our findings substantially expand the known distribution of porcine-associated MRSA ST398 in China to six provinces, thus highlighting its domestic transmission dynamics. This lineage was identified in Qinghai and Shandong (previously reported), as well as in Fujian, Zhejiang, Shanxi, and Guangdong (identified in this study). Given these findings, we strongly advocate for enhanced nationwide surveillance of livestock-associated MRSA ST398, particularly focusing on: 1) Enhanced surveillance of livestock-associated MRSA ST398 in pork production; 2) Zoonotic transmission risk assessment at human-animal interfaces; 3) Genomic epidemiology to track interprovincial and international transmission routes in pork supply chain.

## CRediT authorship contribution statement

**Mingquan Cui:** Writing – review & editing, Writing – original draft, Project administration, Investigation, Funding acquisition, Formal analysis, Data curation, Conceptualization. **Hejia Wang:** Writing – review & editing, Funding acquisition. **Ningning Han:** Writing – review & editing, Resources, Funding acquisition. **Wenli Tang:** Writing – review & editing. **Xiangbin Song:** Writing – review & editing.

## Funding

This work was supported by 10.13039/501100012166National Key Research and Development Program of China (2023YFD1800903–3 and 2022YFD1800400) and Integrated Research on Quality and Safety Control Technologies for Livestock and Poultry Products in the Third Batch of “Key Public Welfare Projects in the Veterinary Drug Industry” by China Institute of Veterinary Drug Control (GY202408).

## Declaration of competing interest

The authors declare no conflict of interest.

## Data Availability

The whole-genome sequencing data of MRSA presented in this study can be found in online repositories https://www.ncbi.nlm.nih.gov/. The names of the repository/repositories and accession number was PRJNA 1240852.

## Appendix A Supplementary data

Supplementary material Image 1

## References

[1] Borg M.A., Camilleri L. (2021). What is driving the epidemiology of methicillin-resistant *Staphylococcus aureus* infections in Europe?. Microb. Drug Resist..

[2] Zarfel G., Krziwanek K., Johler S., Hoenigl M., Leitner E., Kittinger C., Masoud L., Feierl G., Grisold A.J. (2013). Virulence and antimicrobial resistance genes in human MRSA ST398 isolates in Austria. Epidemiol. Infect..

[3] Voss A., Loeffen F., Bakker J., Klaassen C., Wulf M. (2005). Methicillin-resistant *Staphylococcus aureus* in pig farming. Emerg. Infect. Dis..

[4] Ferber D. (2010). Infectious disease. From pigs to people: the emergence of a new superbug. Science.

[5] Jiang N., Wyres K.L., Li J., Feßler A.T., Krüger H., Wang Y., Holt K.E., Schwarz S., Wu C. (2021). Evolution and genomic insight into methicillin-resistant *Staphylococcus aureus* ST9 in China. J. Antimicrob. Chemother..

[6] Kinross P., Petersen A., Skov R., Van Hauwermeiren E., Pantosti A., Laurent F., Voss A., Kluytmans J., Struelens M.J., Heuer O., Monnet D.L. (2017). European human LA-MRSA study group. Livestock-associated meticillin-resistant *Staphylococcus aureus* (MRSA) among human MRSA isolates, European Union/European Economic Area countries, 2013. Euro. Surveill..

[7] Li W., Liu J.H., Zhang X.F., Wang J., Ma Z.B., Chen L., Zeng Z.L. (2018). Emergence of methicillin-resistant *Staphylococcus aureus* ST398 in pigs in China. Int. J. Antimicrob. Agents.

[8] Sun C., Chen B., Hulth A., Schwarz S., Ji X., Nilsson L.E., Ma S., Sun Q., Bi Z., Wang Y., Bi Z., Wu C., Börjesson S. (2019). Genomic analysis of *Staphylococcus aureus* along a pork production chain and in the community, Shandong Province, China. Int. J. Antimicrob. Agents.

[9] Cui M., Ali T., Li J., Song L., Shen S., Li T., Zhang C., Cheng M., Zhao Q., Wang H. (2022). New clues about the global MRSA ST398: emergence of MRSA ST398 from pigs in Qinghai, China. Int. J. Food Microbiol..

[10] CLSI (2018).

[11] Bankevich A., Nurk S., Antipov D., Gurevich A.A., Dvorkin M., Kulikov A.S., Lesin V.M., Nikolenko S.I., Pham S., Prjibelski A.D., Pyshkin A.V., Sirotkin A.V., Vyahhi N., Tesler G., Alekseyev M.A., Pevzner P.A. (2012). SPAdes: a new genome assembly algorithm and its applications to single-cell sequencing. J. Comput. Biol..

[12] Chen L., Zheng D., Liu B., Yang J., Jin Q. (2016). VFDB 2016: hierarchical and refined dataset for big data analysis--10 years on. Nucleic Acids Res..

[13] Zankari E., Hasman H., Cosentino S., Vestergaard M., Rasmussen S., Lund O., Aarestrup F.M., Larsen M.V. (2012). Identification of acquired antimicrobial resistance genes. J. Antimicrob. Chemother..

[14] Inouye M., Dashnow H., Raven L.A., Schultz M.B., Pope B.J., Tomita T., Zobel J., Holt K.E. (2014). SRST2: rapid genomic surveillance for public health and hospital microbiology labs. Genome. Med..

[15] Zhou W., Li X., Osmundson T., Shi L., Ren J., Yan H. (2018). WGS analysis of ST9-MRSA-XII isolates from live pigs in China provides insights into transmission among porcine, human and bovine hosts. J. Antimicrob. Chemother..

[16] Chen H., Yin Y., Li X., Li S., Gao H., Wang X., Zhang Y., Liu Y., Wang H. (2020). Whole-genome analysis of livestock-associated methicillin-resistant Staphylococcus aureus sequence type 398 strains isolated from patients with bacteremia in China. J. Infect. Dis..

[17] Treangen T.J., Ondov B.D., Koren S., Phillippy A.M. (2014). The harvest suite for rapid core-genome alignment and visualization of thousands of intraspecific microbial genomes. Genome Biol..

[18] Argudín M.A., Tenhagen B.A., Fetsch A., Sachsenröder J., Käsbohrer A., Schroeter A., Hammerl J.A., Hertwig S., Helmuth R., Bräunig J., Mendoza M.C., Appel B., Rodicio M.R., Guerra B. (2011). Virulence and resistance determinants of German *Staphylococcus aureus* ST398 isolates from nonhuman sources. Appl. Environ. Microbiol..

[19] Huang Y.C., Chen C.J. (2020). Detection and phylogeny of *Staphylococcus aureus* sequence type 398 in Taiwan. J. Biomed. Sci..

[20] Price J.R., Cole K., Bexley A., Kostiou V., Eyre D.W., Golubchik T., Wilson D.J., Crook D.W., Walker A.S., Peto T.E.A., Llewelyn M.J., Paul J. (2017). Modernising medical microbiology informatics group. Transmission of *Staphylococcus aureus* between health-care workers, the environment, and patients in an intensive care unit: a longitudinal cohort study based on whole-genome sequencing. Lancet Infect. Dis..

