# Supplementary material for: The alert for emergence of pig-associated MRSA ST 398 in multi-regions China
Source: One Health. 2025 Jul 17;21:101132. doi: 10.1016/j.onehlt.2025.101132 (PMC12296544; doi:10.1016/j.onehlt.2025.101132)
Supplement: Supplementary file 1 — Supplementary material [file mmc1.docx]

**Supplementary data**

**Table S1. Metadata for 32 additional MRSA ST398 isolates with different host sources publicly available on NCBI.**

| **MRSA Isolate** | **Geographic region** | **Host** | ***spa* Type** | **SCC*mec* Type** | **MLST** | **Genebank Accession** |
| --- | --- | --- | --- | --- | --- | --- |
| S10 | China | Swine | t011 | V | ST398 | JAMLAV000000000 |
| S15 | China | Swine | t011 | V | ST398 | JAMLAQ000000000 |
| S21 | China | Swine | t011 | V | ST398 | JAMLAK000000000 |
| DY77 | China | Swine | t437 | XII | ST398 | PTAG00000000 |
| 11P8 | Netherlands | Swine | t1456 | V | ST398 | JJDL00000000 |
| C1655 | Spain | Swine | t1255 | V | ST398 | JIZQ00000000 |
| GD5 | China | Human | t034 | V | ST398 | CP019592 |
| FY22 | China | Human | t034 | V | ST398 | NXFU00000000 |
| FY20 | China | Human | t034 | V | ST398 | NTMC00000000 |
| SR389 | China | Human | t034 | V | ST398 | PDFA00000000 |
| SR411 | China | Human | t1255 | V | ST398 | PDFB00000000 |
| S0385 | Australia | Human | t011 | V | ST398 | AM990992 |
| 08BA02176 | Canada | Human | t034 | V | ST398 | NC_18608 |
| 14_11MN_17_08 | Netherlands | Human | t011 | V | ST398 | JJCT00000000 |
| VET0051R | Netherlands | Human | t1184 | V | ST398 | JIVF00000000 |
| 1110700610 | Netherlands | Human | t2329 | V | ST398 | JJEC00000000 |
| RIVM3897 | Netherlands | Human | t034 | UT | ST398 | CP013621 |
| RIVM1607 | Netherlands | Human | t011 | V | ST398 | CP013619 |
| 2010_60_6511_10 | Netherlands | Bovine | UT | IV | ST398 | JJCG00000000 |
| 2010_60_6511_5 | Netherlands | Bovine | t011 | IV | ST398 | JJCE00000000 |
| 2011_60_2078_5 | Netherlands | Bovine | t011 | IV | ST398 | JJCB00000000 |
| FP_N239 | Netherlands | Bovine | t899 | IV | ST398 | JIYQ00000000 |
| DICM09_01587 | Spain | Sheep | t899 | V | ST398 | JIYR00000000 |
| 2009_60_561_1 | Germany | Chicken | UT | V | ST398 | JJCK00000000 |
| Chi_10 | Austria | Chicken | t011 | V | ST398 | JIYV00000000 |
| S56_POEL | Belgium | Chicken | UT | IV | ST398 | JIWS00000000 |
| Tur_22 | Italy | Chicken | t011 | V | ST398 | JIVY00000000 |
| Rd.3 | Germany | Bovine | t899 | IV | ST398 | JIWZ00000000 |
| J01 | China | Human | t011 | V | ST398 | SRR9046751 |
| J12 | China | Human | t034 | V | ST398 | SRR9046752 |
| R09 | China | Human | t034 | V | ST398 | SRR9046749 |
| X05 | China | Human | t034 | V | ST398 | SRR9046750 |

“UT” indicates “untypeable”
